# Eco-Friendly Polyhydroxybutyrate Composite Films Reinforced with Cellulose and Holocellulose Fibers by the Solvent Casting

**DOI:** 10.3390/polym18080997

**Published:** 2026-04-20

**Authors:** Erol Imren, Engin Kocatürk, Ferhat Şen, Mustafa Zor, Şeyma Özlüsoylu, Özge Özgürlük, Deniz Aydemir

**Affiliations:** 1 Department of Forest Industrial Engineering, Bartın University, Bartın 74100, Türkiye; seymaozsoylu@gmail.com (Ş.Ö.); ozgeozgurluk@bartin.edu.tr (Ö.Ö.); denizaydemir@bartin.edu.tr (D.A.); 2 Department of Nanotechnology Engineering, Zonguldak Bülent Ecevit University, Zonguldak 67100, Türkiye; enginkocaturkk96@gmail.com (E.K.); mustafa.zor@beun.edu.tr (M.Z.)

**Keywords:** polyhydroxybutyrate, α-cellulose, holocellulose, bio-based polymer composites

## Abstract

The use of cellulosic reinforcement fillers, including cellulose and holocellulose, in the development of sustainable biopolymer composites has become increasingly essential and continues to attract significant attention in the composite industry. This study aimed to improve the structural and morphological characteristics of the polyhydroxybutyrate (PHB) matrix by incorporating cellulosic fillers—namely, α-cellulose and holocellulose produced via a green processing method—and to evaluate the effect of hemicellulose, present in holocellulose and exhibiting compatibilizing capability, on the overall performance of PHB-based blends. For this, the PHB matrix was first dissolved in chloroform, after which the cellulosic fillers were incorporated into the PHB–chloroform mixtures at 1 wt.% to provide the best homogeneous fiber dispersion. The PHB and cellulosic filler mixtures were blended at 500 rpm with a magnetic mixer for 30 min, and the resulting composite was cast onto a Teflon plate. Scanning electron microscopy (SEM), X-ray diffraction (XRD), and Fourier transform infrared (FTIR) spectroscopy were used to characterize the morphological and structural analysis of the obtained biopolymer-based composites. Thermogravimetric analysis (TG-DTG) was used to determine the thermal properties. The results obtained confirmed the presence of cellulosic fillers in the PHB matrix using FTIR, XRD, and SEM. In contrast to holocellulose, α-cellulose in the PHB matrix was shown to create a more organized structure. Both α-cellulose and holocellulose reinforcements were found to have similar effects on the thermal properties of the PHB matrix. Compared with neat PHB, the amount of residual char was found to be more than 36-fold in the sample containing α-cellulose and more than 41-fold in the sample containing holocellulose.

## 1. Introduction

PHB, the most well-known member of the polyhydroxyalkanoate (PHA) family, is one of the most extensively studied biopolymers and can accumulate up to 80% of the cell dry weight in both natural and recombinant microorganisms [1,2,3]. PHB exhibits inherent brittleness, limiting its processability and practical applications due to its high crystallinity and limited thermal stability. To improve mechanical properties, thermal stability, and printability, several innovations, such as incorporating reinforcing agents, such as cellulose fillers or blending PHB with other biodegradable polymers, such as PLA or polycaprolactone (PCL), were conducted [2,4,5].

Cellulose represents about 33% of total plant biomass and serves as the primary structural component of plant cell walls. It accounts for roughly 50% of wood and nearly 90% of cotton. Several forms of cellulose occur in nature, among which α-cellulose exhibits the highest degree of polymerization and the most remarkable chemical stability [6,7]. α-Cellulose is a white, odorless, and tasteless powder that is insoluble in water. It is a widely used raw material in the production of propellants, paper, paperboard, fabric, electrical cable insulators, and cellulose derivatives, among others [8,9]. Hemicellulose, a lower-molecular-weight polysaccharide associated with cellulose, constitutes the other major component of the polysaccharide fraction in lignocellulosic materials such as wood and straw. Holocellulose refers to the combined content of cellulose and hemicellulose in these materials. It is typically produced through a mild delignification process that preserves the molar mass and fibrillar structure of cellulose while maintaining a high hemicellulose content [10]. One of the primary functions of hemicellulose is to keep together biopolymers such as cellulose and lignin, acting as a natural compatibilizer within the plant cell wall matrix. Its unique ability to connect hydrophilic (cellulose) and hydrophobic (lignin) components makes it an effective natural coupling agent [11,12,13]. This characteristic also highlights its potential role as a natural compatibilizer in polymer composite systems [14,15]. Although biodegradable polymers remain promising, their use is still quite limited compared to traditional plastics. This is due to higher production costs and technological limitations in terms of process ability and functional performance [16]. In the literature, cellulose-based reinforcements are recognized as one of the most ideal reinforcement materials for PHB due to their superior properties, including renewability, biodegradability, thermal stability, low density, high specific strength and hardness, non-toxicity, and corrosion resistance [3,9,17,18]. The incorporation of cellulose reinforcements (e.g., CNF, CNC) into PHB can reduce production costs, a significant obstacle to its widespread marketing [19]. A review of the literature reveals that various forms of cellulose, including cellulose nanocrystals, nanofibers, and microfibrils, as well as hemicellulose, have attracted increased interest in combination with PHB due to the growing demand for environmentally friendly materials. In this context, the combination of PHB with natural polysaccharides such as cellulose and hemicellulose has attracted significant attention for the development of sustainable composite materials. In a study done by D’Arienzo et al. [20], PHB composites reinforced with cellulose fillers exhibited limited improvements in mechanical properties due to weak interfacial adhesion despite good filler dispersion. Similarly, Schmidt et al. researched the effects of cellulose nanocrystals on the properties of PHB composites [21]. The results demonstrated that the incorporation of cellulose nanocrystals derived from paper industry waste at 1 wt% and 3 wt% led to optimal dispersion at low concentrations, significantly enhancing the mechanical and thermal properties of PHB composites. In another study, Zhang et al. studied PHB/PHBV-based nanocomposites containing both cellulose nanocrystals (CNCs) and nanofibers (CNFs) [22]. The results showed that adding CNF nearly doubled Young’s modulus and improved tensile strength. Avecilla-Ramírez found that, in a study using luffa fibers containing approximately 60% cellulose and 15% hemicellulose, the mechanical and morphological performance of the resulting PHB composites improved as the fiber content increased [23]. In a study by Mendes et al., a blend of hemicellulose and PHB (90:10 wt/wt) with acetic acid exhibited an optimal melting temperature and crystallinity due to strong interactions with the hemicellulose hydroxyl groups [18]. A higher hemicellulose loading ratio generally decreased the mechanical properties of the blends. This study indicated that cellulose- and hemicellulose-based polysaccharides at low contents hold strong potential to enhance the mechanical, thermal, and environmental performance of PHB composites. However, interfacial compatibility, filler dispersion, filler ratio, and processing parameters remain critical factors that directly influence the overall performance, emphasizing the need for further systematic studies.

Although numerous studies have examined the incorporation of α-cellulose and cellulosic fibers into poly (hydroxybutyrate) (PHB) matrices, these fillers have typically been obtained via conventional extraction methods using hazardous chemicals. Moreover, the comparative effects of holocellulose and α-cellulose isolated under identical processing conditions have not been systematically clarified. In particular, the specific contribution of the hemicellulose-rich, branched, and predominantly amorphous fraction present in holocellulose to the molecular organization, crystallization behavior, and thermal stability of PHB remains insufficiently understood. Therefore, this study aims to isolate holocellulose and α-cellulose using an environmentally friendly deep eutectic solvent (DES) system under identical processing conditions and to incorporate both fillers into the PHB matrix at an equal loading level (1 wt%). By eliminating variability arising from different extraction routes and filler concentrations, the work seeks to directly reveal the intrinsic structural and thermal influences of each cellulosic fraction on PHB. The originality of this study lies in its controlled comparative design, which enables the clarification of the distinct role of hemicellulose-containing holocellulose, and in the evaluation of how hemicellulose present in holocellulose, acting as a compatibilizing component, influences the chain organization and crystallinity of PHB. To achieve this objective, the structural and interfacial characteristics of the resulting biocomposites were analyzed using FTIR, XRD, and SEM. At the same time, their thermal behavior and stability were comprehensively evaluated through TG–DTG analyses.

## 2. Materials and Methods

### 2.1. Materials

In this study, polyhydroxybutyrate granules with a molecular weight of 190 kDa were supplied from Bulk Reef Supply (Golden Valley, MN, USA). Chloroform (99%), used to dissolve the PHB granules, was purchased from Sigma Aldrich (St. Louis, MO, USA). The stone Pine (*Pinus pinea* L.) cones used to obtain cellulose materials, including α-cellulose and hemicellulose, were collected in Izmir, Turkey. The samples were ground using a Wiley mill and passed through a Retsch AS 200 sieve shaker (Retsch GmbH, Haan, Germany). The fraction retained on a 60-mesh sieve was used in the experiments. Green solvents used for the preparation of DESs (a mixture of choline chloride and lactic acid) were purchased from Sigma-Aldrich (St. Louis, MO, USA). Cellulose and holocellulose fibers were used in biopolymer composites due to their natural, renewable, and fully biodegradable structures, which enhance environmental compatibility and support the overall degradability of the material in natural conditions.

### 2.2. Preparation of PHB Biocomposites with α-Cellulose and Holocellulose

The deep eutectic solvent (DES) was prepared using lactic acid as the hydrogen-bond donor (HBD) and choline chloride as the hydrogen-bond acceptor (HBA). The molar ratio of ChCl to lactic acid was set at 1:9 (w/w). Accurately weighed amounts of solid choline chloride and liquid lactic acid were placed in a round-bottom flask and heated on a magnetic stirrer with temperature control to approximately 80 °C under continuous stirring (300–500 rpm) until a transparent, homogeneous liquid phase was obtained (approximately 1–2 h). The resulting solution was cooled to room temperature and stored in sealed bottles until further use. For the treatment process, the prepared DES was applied to the woody biomass at a solid-to-liquid ratio of 1:15, and the mixture was heated under microwave irradiation at 150 °C for 50 min in a microwave reactor (Milestone Ethos, Sorisole, Italy). Woody samples were extracted with ethanol in accordance with TAPPI T204 [24]. The holocellulose content of woody samples was determined using the chlorite method initially developed by Wise and Karl (1962) [25]. The α-cellulose content was determined from the holocellulose fraction according to the TAPPI T203 OS-71 standard method [26]. The contents of holocellulose, α-cellulose, and hemicellulose in woody samples are presented in Table 1. Hemicellulose content in the samples was determined by subtracting α-cellulose from holocellulose. Diameters and the light of both α-cellulose and holocellulose were similar at 10–20 µm and up to 50–100 µm.

Amounts of PHB, α-cellulose, and holocellulose specified in Table 2 were placed in a beaker, and then 70 mL of chloroform was added to the a beaker. The mixture was continuously stirred in an experimental setup included a magnetic and ultrasonic mixer for 2 h at approximately 62 °C (the boiling point of chloroform). The mixture was also placed in an ultrasonic bath for 30 min. to ensure a homogeneous distribution of α-cellulose and holocellulose at varying mass ratios. The resulting homogeneous mixture was poured into a Petri dish, and the chloroform was degassed under vacuum in a fume hood at room temperature to obtain cellulose-added biocomposites. A cellulosic filler loading of 1 wt% was selected to ensure homogeneous dispersion within the PHB matrix while minimizing fiber agglomeration and stress concentration effects commonly observed at higher loadings. Previous studies have demonstrated that low cellulose contents (≤1–3 wt%) in solvent-cast PHB/PHBV systems promote improved interfacial interaction and controlled crystallization without compromising film integrity or inducing brittleness [27,28,29]. Higher filler contents (>3 wt.) frequently lead to particle clustering and heterogeneous morphology, negatively affecting mechanical and barrier performance [12]. Therefore, 1 wt% represents a balanced formulation that maximizes dispersion quality while preserving the structural continuity of the PHB matrix.

### 2.3. Characterization and Measurements

#### 2.3.1. Physical Properties

Densities of the PHB-based composites were measured using the water-displacement method according to ASTM D792-13. Water absorption and thickness swelling ratios were determined after 24 h of water exposure. The density determination were conducted on six replicates for each formulation, and the averages were used.

#### 2.3.2. FT-IR Spectroscopy

A Shimadzu IRAffinity-1S FTIR spectrometer, equipped with a germanium crystal ATR attachment (Shimadzu Corporation, Kyoto, Japan), was used to examine the chemical structure of the samples. With a resolution of 2 cm^−1^, the measurements were carried out from 4000 cm^−1^ to 700 cm^−1^ spectral range.

#### 2.3.3. X-Ray Diffraction Analysis

XRD analysis was conducted using a RIGAKU SmartLab X-ray diffractometer (Tokyo, Japan). The measurements were performed with CuKα radiation (λ = 1.54 Å) at 45 kV and 40 mA, over a 2θ range of 10° to 90°. The crystallinity index (CI) was calculated using the following formula:

$${CI}_{\%}=\frac{\sum A_{c}}{\sum \left(A_{c}+A_{a}\right)}$$

where CI, expressed as a percentage, was derived by comparing the integrated area of the crystalline peaks (A_c_) to the area of the amorphous halo (A_a_).

#### 2.3.4. Thermogravimetric (TG-DTG) Analysis

TGA analysis was performed using a TG/DTG Hitachi STA 7300 analyzer (Tokyo, Japan) under nitrogen (N_2_) atmosphere. At a rate of 10 °C/min, the samples were heated from room temperature to 750 °C.

#### 2.3.5. Scanning Electron Microscopy (SEM)

Using a Tescan MAIA3 XMU SEM (Brno, Czech Republic), surface (morphology) characterization was carried out on the fracture surfaces of the samples at different magnifications. A Quorum Q150T ES sputter coater (Quorum Technologies, Lewes, East Sussex, UK) was used to apply a thin layer of a mixture of gold-platinum to all samples before morphological characterization to enhance electron flow.

## 3. Results and Discussion

### 3.1. Physical Properties of PHB Biocomposites

PHB biocomposites were produced by incorporating several cellulosic fillers, including alpha cellulose, holocellulose, and their combination, and their density, water absorption, and thickness swelling values were investigated. The obtained results were presented in Table 3.

As seen in Table 3, it is observed that while the density of neat PHB is 1.23 g/cm^3^, the incorporation of 1% α-cellulose (AS), holocellulose (HOLO), and α-cellulose + holocellulose (AS-HOLO) slightly decreased the density values of 1.19, 1.18, and 1.18 g/cm^3^, respectively. This slight decrease may be attributed to the relatively lower density of cellulose-based fibers compared to the PHB matrix and their potential to induce microstructural void formation within the matrix even at low filler loadings. Previous studies have reported that low-level cellulose reinforcement may cause limited agglomeration and interfacial voids within polymer matrices, leading to a partial reduction in density [30]. Furthermore, holocellulose containing hemicellulose may exhibit a more heterogeneous distribution within the PHB matrix, which could contribute to a more pronounced decrease in density.

Regarding water absorption, neat PHB exposed to water for 24 h absorbed 1.6%, whereas the addition of AS and AS-HOLO increased water uptake. The increase is more pronounced in HOLO-containing composites, likely due to the presence of hemicellulose. Although PHB has a semi-crystalline structure, the incorporation of cellulosic materials increases the hydrophilicity of the biocomposites because cellulose and hemicellulose possess hydrophilic hydroxyl groups. In particular, holocellulose exhibits a more amorphous, hydrophilic character due to its hemicellulose content, leading to higher water absorption than α-cellulose. In terms of thickness swelling, neat PHB exhibited 2.4% swelling, whereas the addition of α-cellulose and holocellulose led to higher thickness swelling values. The tendency of cellulosic fibers to swell upon water absorption and the resulting stress generation at the matrix–fiber interface may adversely affect the dimensional stability of the composites. Mainly because of the higher amorphous fraction of holocellulose, its greater water-uptake capacity appears to contribute to increased thickness swelling. Overall, it has been determined that even at a low cellulose loading of 1%, noticeable changes occur in water absorption and thickness swelling behavior.

### 3.2. Chemical Properties of PHB Biocomposites

**Figure 1:**
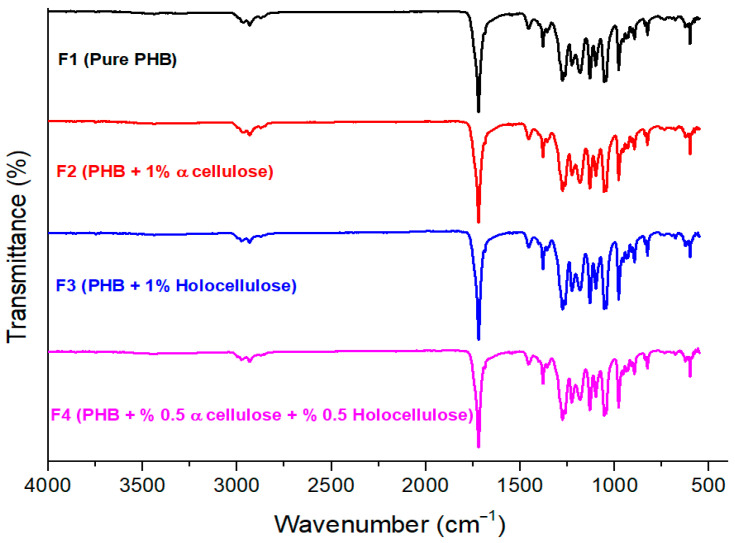
FTIR spectra of cellulose derivative reinforced PHB biocomposites.

Different functional groups in neat PHB and PHB composites reinforced with cellulose derivatives were identified by FTIR spectroscopy. FTIR spectra of the samples were measured over the range 4000–550 cm^−1^. A comparison of the FTIR spectra obtained in this study (Figure 1) with those reported in the literature showed strong consistency. Previous research clearly indicated that the characteristic FTIR band of PHB originated from the stretching vibration of the C=O group, which typically appears at around 1721 cm^−1^ [31,32,33]. In addition, the crystallinity phase of PHB was shown at the peaks observed at 816–826 cm^−1^, 1276–1278 cm^−1^, and 1721 cm^−1^. According to the spectra, the absorption bands at 2939 and 2880 cm^−1^ correspond to the asymmetric stretching vibrations of methyl and methylene groups, respectively [32,33]. These findings were consistent with previously reported PHB spectra, confirming the presence of PHB in the samples. In addition, FTIR results showed that the cellulose-derived fillers and their combinations (F2, F3, and F4) exhibited similar spectral features across all samples, which can be attributed to the homogeneous structure of the prepared composites. Overall, the spectra indicated that the incorporation of α-cellulose and holocellulose did not cause any significant shifts or changes in the FTIR spectra of the PHB matrix.

### 3.3. XRD Analysis of PHB Biocomposites

**Figure 2:**
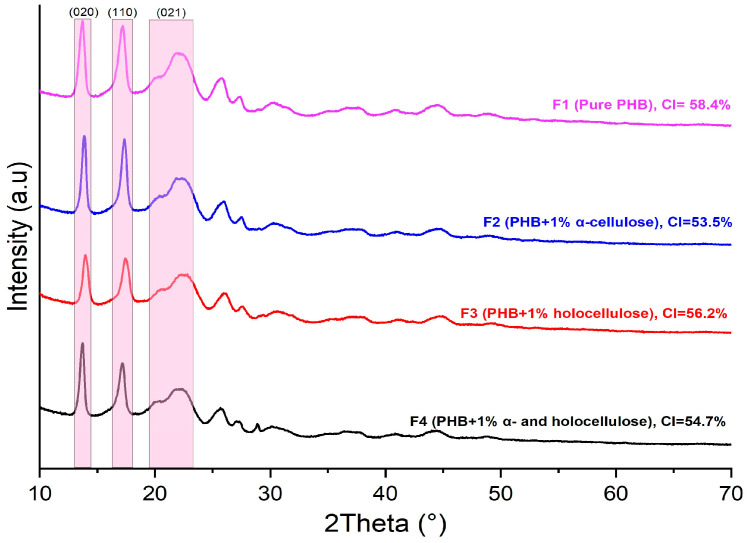
XRD graph of cellulose derivative reinforced PHB biocomposites.

Crystal structure analysis of pure PHB (F1), α-Cellulose-reinforced PHB (F2) (1%), holocellulose-reinforced PHB (F3) (1%), and both α-Cellulose- (0.5%) and holocellulose-reinforced PHB (F4) (0.5%) biocomposites was carried out via XRD. The XRD patterns of the composite films were examined between 10° and 90°, and the diffractograms obtained with assigned planes are shown in Figure 2. The main XRD peak positions, corresponding crystal planes, and crystallinity index (CI) values of all samples are summarized in Table 4. As seen in the XRD results, pure PHB (F1) exhibited the typical α-type structure due to melting and crystallization. Previous studies have reported that PHB crystallizes in an orthorhombic lattice structure [34,35]. The diffractograms of PHB and α-Cellulose/holocellulose-reinforced PHB (F1, F2, F3, and F4) exhibited similar profiles corresponding to the orthorhombic unit cell normally obtained for pure PHB (F1) [36,37]. In addition to a less intense peak (2θ ≈ 20°) tuned to the (021) plane, the biocomposites also displayed two strong crystalline peaks at 2θ ≈ 13.5° and 17° assigned to the (020) plane and (110) plane of the orthorhombic unit cell. This suggests that the biocomposites contained a small amount of orthorhombic β-form crystal with zigzag conformation in addition to the orthorhombic α-form crystal with helical chain conformation, which is the most common crystal structure of the PHB molecule. A greater degree of molecular strain in the amorphous areas between the α-crystalline lamellae is indicated by the occurrence of β-form crystals [36,38,39]. According to Figure 2, the α-cellulose-reinforced PHB composite (F2) showed a stronger intensity of the peak corresponding to the (020) crystal plane than the holocellulose-reinforced PHB (F3), suggesting the presence of a preferred orientation and increased crystallinity. According to published research, adding cellulose to PHB as a nucleator speeds up the crystallization of PHB composites [38]. This finding suggests that, at the same loading level, α-cellulose promoted a more ordered crystalline structure in the PHB matrix than holocellulose. Overall, the structural integrity of the cellulose-reinforced PHB composites is clearly supported by the strong agreement between the FTIR and XRD results.

XRD analyses revealed that pure PHB exhibited the highest crystallinity index (CI = 58.4%), consistent with the typical crystallinity range reported in the literature. The incorporation of α-cellulose significantly reduced the CI value (53.5%), indicating that it is the filler phase that most strongly disrupts the orderly packing of PHB chains; this behavior is in agreement with the findings on PHB–cellulose interactions reported in studies [7,22] and Ref. [42]. In contrast, holocellulose, due to its more amorphous character, showed better compatibility with the matrix and resulted in a more limited reduction in crystallinity (CI = 56.2%), thus exerting a less pronounced impact on PHB crystalline phases. The CI value of the F4 formulation (54.7%), which contains both α-cellulose and holocellulose, indicated that the combination of the two fillers produces a moderate decrease in crystallinity. Similar results reported in studies [11,18] and Ref. [25] suggested that this behavior arises from a “balance effect” typical of dual-phase lignocellulosic systems. Overall, the variations in CI values clearly demonstrated the influence of filler type on the matrix compatibility, confirming that the structural regularity of PHB was most strongly disrupted by α-cellulose.

### 3.4. Thermal Properties of PHB Biocomposites

**Figure 3:**
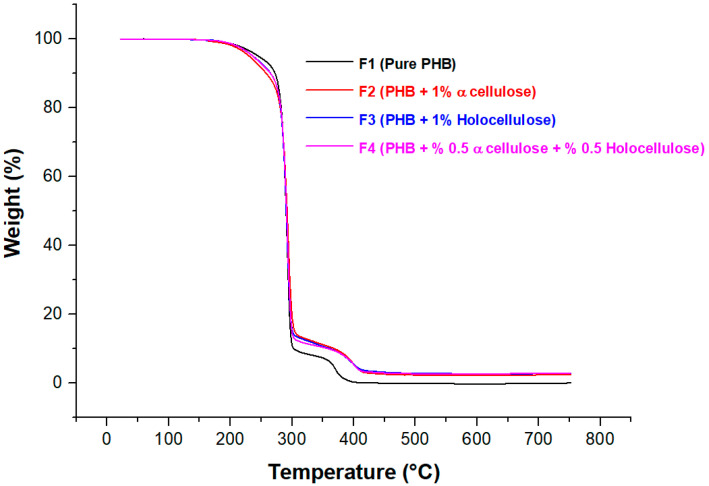
TGA curves of cellulose derivative reinforced PHB biocomposites.

**Figure 4:**
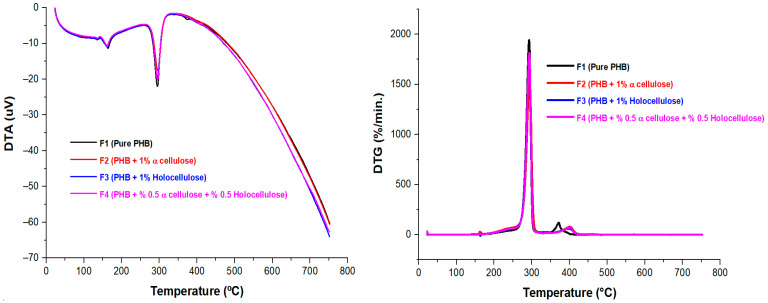
DTG of PHB-based biocomposites.

The thermal behavior of the biocomposites was evaluated using TG and DTG curves, as shown in Figure 3 and Figure 4 and summarized in Table 5. According to the TGA results, pure PHB exhibited a curve similar to that observed in previous studies, as reported in [9] and [42]. Thermogravimetric curves for both pure PHB and the biocomposite samples containing α-cellulose and holocellulose additives showed weight loss in two steps. In addition, the DTG curves in Figure 4 showed a small initial peak at around 150 °C that is mainly associated with moisture loss. About 250–300 °C was the temperature at which the first deterioration step took place, and between 300 and 400 °C was the temperature at which the second degradation step took place. The low temperatures observed in the first degradation step of the biocomposites may have led to premature degradation of cellulose derivatives within the matrix, and since bio-based materials were used, the initial mass loss was likely due to moisture. This premature degradation of cellulose derivatives subsequently accelerated PHB degradation. Due to random ester bond cleavage, the primary mechanism of PHB thermal breakdown was the β-elimination of PHB chains, which promotes the formation of crotonic acid, dimeric, trimeric, and tetrameric volatiles. This process is known to occur primarily at temperatures above 200 °C [43,44]. The second degradation was due to thermal degradation of the PHB-based biocomposites’ main polymer chains. The main degradation occurred at approximately 290–300 °C, and at this stage, the biocomposite samples lost approximately 80–90% of their mass. Based on this, it can be said that α-cellulose and holocellulose did not have a significant effect on the main degradation temperature. It was observed that the maximum degradation temperature of PHB increased with the addition of α-cellulose and holocellulose. Based on this, it can be said that the produced biocomposites containing cellulose derivatives exhibit higher thermal stability than pure PHB. According to earlier research, the filler’s efficient dispersion within the matrix was responsible for the enhanced thermal stability [45,46]. The increased thermal stability observed with the addition of α-cellulose and holocellulose to the PHB matrix indicates that the resulting composite structures exhibit a more crystalline morphology than pure PHB, consistent with the XRD findings. Given the high temperatures used to produce biodegradable films and molded parts in extrusion and injection molding, it is important to accept high decomposition temperatures because the material must be durable and resist structural degradation; this is an important factor in polymer processing in industry [47,48]. Generally, biocomposites exhibited a small amount of residual material consisting of ash and carbon. All biocomposites had an exceptionally high residue content compared to pure PHB. Compared with pure PHB, the PHB composite reinforced with 1% α-cellulose exhibited an approximately 36-fold increase in residue content. A similar trend was observed for the holocellulose-reinforced PHB (1%), where the residue content was about 41 times higher (Table 5). Therefore, it can be concluded that the holocellulose addition to the PHB matrix acted as a more protective barrier compared to the α-Cellulose addition. This indicated an improvement in the flame-retardant performance of the cellulose-reinforced biocomposites. The residue structure formed on the surface of the biocomposite during combustion can act as a protective thermal barrier against further combustion. The positive and harmonious interaction between cellulose and the PHB matrix may indicate that it plays a significant role in promoting residue formation during thermal degradation.

The DTG results were consistent with each other. Examination of the DTG curves for the PHB-based biocomposites revealed two distinct thermal peaks in both the pure PHB and the cellulose-derived biocomposites. The distribution of crystal sizes inside PHB, which shows crystal heterogeneity, is responsible for this [46]. The melting point (T_m_) was represented by the first of these peaks, while the decomposition temperature (T_d_) was represented by the second. Accordingly, all composites began to melt in the range of approximately 155–165 °C, and their melting temperatures were very similar. A similar pattern could be observed for the decomposition temperatures. According to the DTG curves, it was determined that pure PHB began to melt at 162.5 °C (T_m_). This result corresponded to almost the same temperatures for the biocomposites. In addition, a endothermic peak was observed as temperatures increased. These peaks indicated that pure PHB and its biocomposites decomposed over the 280–300 °C range.

### 3.5. Morphological Characterization of PHB Biocomposites

**Figure 5:**
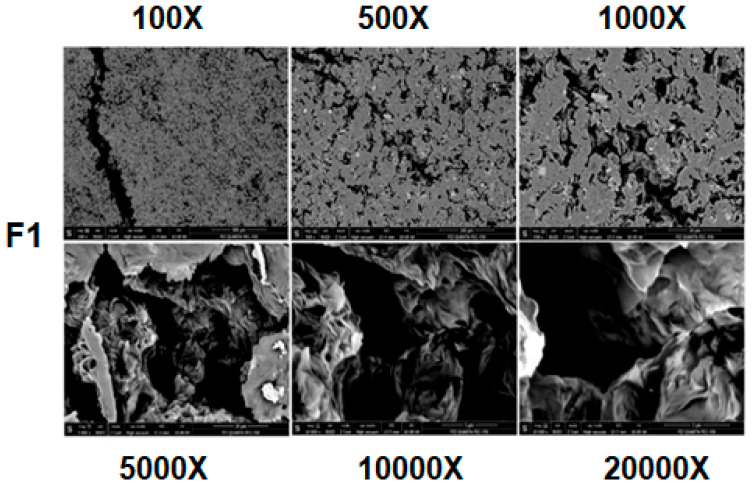
Morphological structure of PHB (100×, 500×, 1000×, 5000×, 10,000×, and 20,000×).

SEM pictures of pure PHB at various magnifications are displayed in Figure 5. PHB’s crystallization behavior is crucial since it dictates a lot of its characteristics, most notably its shape. Typically, PHB is characterized by high crystallinity and a low crystallization rate, and it is expected to develop the characteristic fractures characteristic of brittle polymers (a monophasic, compact, and generally homogeneous surface) [17,19,47].

**Figure 6:**
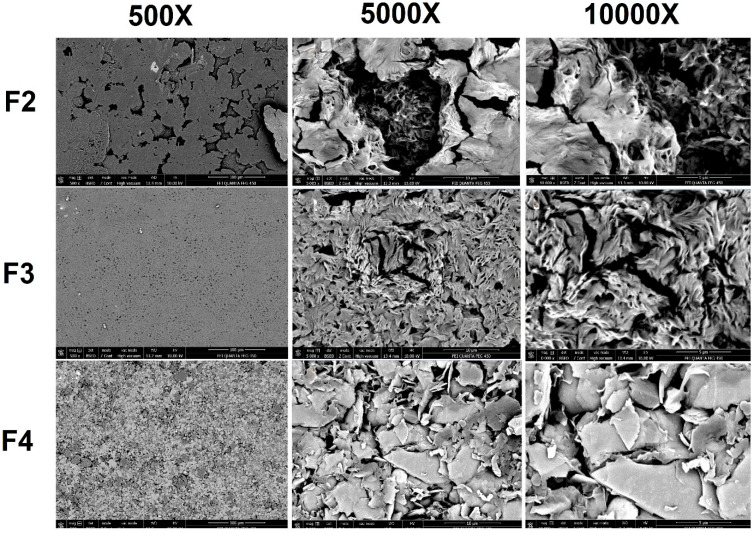
Morphological structure of cellulose derivatives reinforced biocomposites.

Figure 6 shows the SEM images at different zooms. The images showed 1% α-Cellulose (F2), 1% holocellulose (F3), and both α-Cellulose (0.5%) and holocellulose reinforcement (0.5%) (F3) in the composite matrix. When the morphological images of the biocomposite materials were examined, it was observed that the morphological structure of pure PHB changed with the addition of α-cellulose, holocellulose, and their combination. Based on the images, the surface roughness of the biocomposite increased with the addition of cellulosic particles. Compared to the pure PHB morphology, the addition of 1% α-cellulose reduced the amount of voids within the biocomposite and the cell diameters that form the porous structure. Similar results were obtained when 1% holocellulose was added, since there was a larger reduction in the number of voids in the composite than when α-cellulose was added. Based on this, it can be concluded that the addition of holocellulose to the PHB matrix results in a more homogeneous structure. In addition, the additive combination in the matrix improves the structure by forming interfacial bonds between cellulose particles, giving the composite a more homogeneous appearance.

## 4. Conclusions

Despite rapid advancements in science, engineering, and materials technology, the demand for environmentally friendly and high-performance composite systems continues to grow. In this context, the present study successfully developed PHB-based biocomposites reinforced with cellulose-derived fillers, namely α-cellulose and holocellulose, and evaluated their structural and thermal properties. The results demonstrated that the incorporation of these fillers partially achieved the intended modification of the PHB matrix, particularly in terms of thermal behavior and structural characteristics. FTIR analysis indicated the absence of significant chemical interactions between PHB and the cellulosic reinforcements, suggesting that the composites were mainly governed by physical interactions. XRD results revealed that the addition of cellulose-based fillers did not alter the characteristic crystalline pattern of PHB; however, a decrease in crystallinity index was observed. TGA results showed a slight improvement in thermal stability, indicating that the fillers contributed to delaying thermal degradation to a limited extent. SEM characterization showed the changes in the morphological properties of the PHB composites with cellulosic fillers. In addition, the solvent used during composite preparation was removed by evaporation, but no recovery or recycling strategy was implemented, which should be considered in future work from a sustainability perspective. Overall, PHB-based composites reinforced with cellulose-derived fillers represent a promising material system for sustainable applications. However, further studies focusing on mechanical optimization, interfacial compatibility, and process sustainability are necessary to fully exploit their potential. The developed materials can be considered potential candidates for environmentally friendly applications such as sustainable packaging and bio-based consumer products, particularly where moderate thermal stability and biodegradability are required.

## Figures and Tables

**Table 1:** Holocellulose, α-cellulose, and hemicellulose contents of the samples.

Samples	Control (%)	ChCl:9LA (%)
Holocellulose	69.95	63.58
α-cellulose	41.81	50.76
Hemicellulose	28.14	12.82

Alpha cellulose and holocellulose do not contain any lignin impurity, and they also consist of cellulose and hemicellulose fibers.

**Table 2:** Formulation of α-cellulose- and holocellulose-reinforced biocomposites.

Samples	PHB (%)	α-Cellulose (%)	Holocellulose (%)
F1	100	-	-
F2	99	1	-
F3	99	-	1
F4	99	0.5	0.5

**Table 3:** Some physical properties of α-cellulose- and holocellulose-reinforced biocomposites.

Samples	Density (g/cm^3^)	Water Absorption (%)	Thickness Swelling (%)
F1	1.23 (±0.02)	1.6 (±0.2)	2.4 (±0.03)
F2	1.19 (±0.03)	2.1 (±0.1)	2.5 (±0.02)
F3	1.18 (±0.02)	2.3 (±0.3)	2.9 (±0.03)
F4	1.18 (±0.04)	2.2 (±0.1)	2.8 (±0.02)

**Table 4:** Main XRD peak positions, corresponding crystal planes and crystallinity index (CI) values of cellulose derivative reinforced PHB biocomposites.

Samples	2θ (°)	Crystal Plane (hkl)	CI (%)
F1	13.5, 17.0, 20.0	(020), (110), (021)	58.4
F2	13.5, 17.0, 20.0, ~22.5	(020), (110), (021), (200)	53.5
F3	13.5, 17.0, 20.0, broad ~22	PHB planes + amorphous contribution	56.2
F4	13.5, 17.0, 20.0, ~22	Combined crystalline + amorphous	54.7
Cellulose [40]	14.6, 16.6, 22.7	($\bar{110}$), (110), (200)	
Holocellulose [41]	14.8,16.5, 22.3	($\bar{110}$), (110), (200)	

**Table 5:** Thermal properties of PHB-based biocomposites.

Samples	T_5%_ (°C)	T_50%_ (°C)	DTG_max_ (°C)	Residue at 750 °C (%)	T_m_ (°C)	T_d_ (°C)
F1	245	290	292	0.07	158	283–298
F2	230	292	294	2.52	159	285–295
F3	237	291	293	2.87	158	285–300
F4	236	290	293	2.98	160	283–295

## Data Availability

The original contributions presented in this study are included in the article. Further inquiries can be directed to the corresponding authors.

## Abbreviations

The following abbreviations are used in this manuscript:

PHB	Polyhydroxybutyrate
PHA	Polyhydroxyalkanoate
PLA	Polylactic acid
PLC	Polycaprolactone
CNC	Cellulose nanocrystal
CNF	Cellulose nanofiber
FTIR	Fourier transform infrared
XRD	X-ray diffraction
DES	Deep eutectic solvent
